# Computer-aided evaluation of the correlation between MRI morphology and immunohistochemical biomarkers or molecular subtypes in breast cancer

**DOI:** 10.1038/s41598-017-14274-3

**Published:** 2017-10-23

**Authors:** Sen Jiang, You-Jia Hong, Fan Zhang, Yang-Kang Li

**Affiliations:** 1 Department of Radiology, Cancer Hospital of Shantou University Medical College, Guangdong, China; 2 Department of Ultrasound, Cancer Hospital of Shantou University Medical College, Guangdong, China; 3 Oncology Research Laboratory, Cancer Hospital of Shantou University Medical College, Guangdong, China

## Abstract

Studies using tumor circularity (TC), a quantitative MRI morphologic index, to evaluate breast cancer are scarce. The purpose of this study is to evaluate the correlation between TC and immunohistochemical biomarkers or molecular subtypes in breast cancer. 146 patients with 150 breast cancers were selected. All tumors were confirmed by histopathology and examined by 3.0T MRI. TC was calculated by computer-aided software. The associations between TC and patient age, tumor size, histological grade, molecular subtypes, and immunohistochemical biomarkers including estrogen receptor (ER), progesterone receptor (PR), human epidermal growth factor receptor 2 (HER2), and Ki67 were analyzed. TC correlated inversely with tumor size (r = −0.224, P < 0.001), ER (r = −0.490, P < 0.001) and PR (r = −0.484, P < 0.001). However, TC correlated positively with Ki67 (r = 0.332, P < 0.001) and histological grade (r = 0.309, P < 0.001). In multiple linear regression analysis, tumor size, ER, PR and Ki67 were independent influential factors of TC. Compared with HER2-overexpressed (61.6%), luminal A (54.7%) and luminal B (52.3%) subtypes, triple-negative breast cancer (TNBC) showed the highest score of TC (70.8%, P < 0.001). Our study suggests that TC can be used as an imaging biomarker to predict the aggressiveness of newly diagnosed breast cancers. TNBC seems to present as an orbicular appearance when comparing with other subtypes.

## Introduction

Breast cancer is a kind of highly heterogeneous tumor. It had been identified distinct molecular subtypes that vary in clinical outcome, therapeutic responses as well as prognosis by gene expression profiling. Variarion responses and outcome still exist within the same subtype because of the different level of biomarkers expression^1–3^. The 2011 St.Gallen panel^4^ advised using immunohistochemical (IHC) biomarkers including estrogen receptor (ER), progesterone receptor (PR), human epidermal growth factor receptor 2 (HER2), and Ki67 as substitutes defining molecular subtypes. And the four major subtypes luminal A, luminal B, HER2-overexpressed and triple-negative breast cancer (TNBC) were defined. Magnetic resonance imaging (MRI), a noninvasive and high sensitive examination, has been increasingly used in the assessment of breast disease, including the differential diagnosis of benign and malignant lesions, preoperative evaluation, pretreatment planning and efficacy prediction^5,6^. The correlation between the MRI morphology as well as dynamic features and the molecular subtypes of breast cancer have been reported^7,8^. But most previous studies were based on describing the findings using a lexicon by the radiologist subjectivly. Although most of lexicon is accepted generally, it turned to be variable with different observer^6,9^. Quantitative analysis of the MRI dynamic features in breast cancer had been reported^10,11^. Nevertheless, studies using tumor circularity (TC), a quantitative MRI morphologic index based on computer-aided software, to evaluate breast cancer are scarce. The present study aim to evaluate the correlation between TC and IHC biomarkers or molecular subtypes in breast cancer.

## Material and Methods

### Patients

This retrospective study was approved by the institutional review board. The need for informed consent was waived. Between March 2015 and December 2016, 185 women (age range, 28–77 years, mean age 50.0 ± 10.1 years) with pathologically proven breast cancers were selected. Inclusion criteria: (1) no patient received any treatment; (2) each patient had complete breast MRI data; and (3) all tumors had the IHC biomarker data.

### MRI Technique

All breast MRI examinations were performed at 3.0 T (GE medical systems, Discovery MR750) with the patient prone and by using a dedicated eight-channel surface breast coil. The standard imaging protocol included a localizing MRI sequence followed by an axial T2-weighted fat-suppressed sequence, an axial T1-weighted non–fat-suppressed sequence, an axial T1-weighted simultaneous fat-suppressed sequence performed before and six times after a rapid bolus injection, and a conventional contrast-enhanced sagittal T1-weighted fat-suppressed sequence.

For dynamic contrast-enhanced examination, contrast media (Magnevist, Bayer Schering Pharma, Germany) was administered immediately after the end of first (pre-contrast) sequence as a bolus intravenous injection at a dose of 0.1 mmol/kg and at the rate of 3.0 ml/s. All MRI sequences and parameters were listed on Table 1.

**Table 1:** Breast MRI sequences and parameters.

Sequences	TR (ms)	TE (ms)	FOV (mm)	Matrix	Slice thickness (mm)	Slice distance (mm)
T1WI	420	7–41.8	400*400	320*256	5	1
T2WI	5540	85	320*320	320*256	5	1
Axial T1WI enhanced	3.9	1.1	360*360	320*320	1.4	—
Sagittal T1WI enhanced	4.9	1.2	240*240	256*224	1.8	—

### Image analysis

All images were prospectively evaluated by two radiologists with 7 and 5 years of experience, respectively, in MRI imaging of breast tumors. The readers were blinded to the histopathological results. They reviewed the MRI images with the use of the Breast Imaging Reporting and Data System (BI-RADS) lexicon. Lesions were described as mass or non-mass-like enhancement. Non-mass-like enhancement lesions were excluded from our study due to the exhibition of poorly defined boundaries, leading to difficulty in the analysis of morphology^11^. Tumor size was measured on the largest diameter in the post-contrast axial or sagittal section. Then image of this section was digitally transferred from the picture archiving and communications system workstation to a personal computer with image processing software (photoshop, version CS6), which can automatically calculate perimeter and area, as well as TC after profiling the mass. TC was calculated quantitatively and automatically through this software following the equation: TC = 4π*area/perimeter^2^. The score of TC ranged from 0 to 100%. It turned to be more orbicular if getting higher TC score. It means that a perfect circle-shaped tumor had a 100% score of TC. Besides, axillary lymphadenopathy was defined as lymph nodes greater than 10 mm in short axis dimension.

### Histopathologic analysis

From the initial surgical or puncture biopsy specimens, histological type, pathological grade, lymph node status were obtained by pathologist without knowledge of the MRI findings. IHC analysis for ER, PR, HER2 and Ki67 were also verified by the same pathologist. ER and PR status were evaluated using a percentage of positive cells with nuclear staining. The score of each receptor was considered to be positive if the expression was greater than 10% and negative if the expression was less than 10%. HER2 status was scored as -, 1+, 2+ or 3+, using IHC analysis, as well as fluorescence *in situ* hybridization (FISH) if the score performed 2+ for IHC. A positive HER2 result is IHC staining of 3+, or 2+ with a FISH result confirmed gene amplification^12^. The Ki67 index was analyzed as the percentage of positive cells with nuclear staining in average of five high power field. According to the 2015 St.Gallen panel^13^, surrogate molecular subtypes of breast cancer were classified depend on the status of ER, PR, HER2 and the Ki67 index (Table 2).

**Table 2:** Classification of surrogate molecular subtypes of breast cancer.

Subtype	Receptor status and Ki67 index
Luminal A	ER and/or PR positive, HER2 negative, Ki67 ≦ 20%
Luminal B	ER and/or PR positive, HER2 negative, Ki67 ≧ 20%
	ER and/or PR positive, HER2 positive, any Ki67 index
HER2-overexpressed	ER and PR negative, HER2 positive
Triple-negative (TNBC)	ER and PR and HER2 negative

### Data and statistical analysis

MRI data, including tumor size and TC, were recorded as the mean of values measured by two radiologists. Tumor size was stratified into four subgroups (<15 mm, 15–24 mm, 25–35 mm, and >35 mm). Histological type of tumor was classified as two subgroups (infiltrating ductal carcinoma and non-infiltrating ductal carcinoma). Pathological grade of tumor was classified as three subgroups (low, intermediate, and high). Axillary lymph node was classified as two subgroups (positive and negative).

Biomarkers were divided into three groups separately for between-group estimation. The ER and PR scores were divided into negative (<10%), positive (10% to 89%), and strongly positive (≧90%). HER2 was treated as non-expression (negative), low-expression (1+ or 2+ and FISH-negative) and overexpression (3+ or 2+ and FISH-positive). While the Ki67 indices were divided into low (<20%), intermediate (20% to 39%), and high (≧40%).

Intraclass correlation coefficient was used to assess the variability of TC calculation by two radiologists. Pearson’s rand correlation coefficients was used to calculate the pairwise correlations between TC and patient age, tumor size, biomarkers and pathological variables. Further multiple linear regression was use to determine the independent influential factors of TC. Between-group estimates of TC were compared with one-way analysis of variance (ANOVA) following a Bonferroni test not only in the above biomarkers groups, but also among the subtypes.

All analyses were performed using statistics software (SPSS, version 22), and a *P* value < 0.05 was considered to indicate a statistically significant difference.

## Results

146 patients with mass enhancement were found in all 185 patients. While 39 patients with non-mass-like enhancement were excluded from the study because of the poorly defined boundaries, leading to difficulty in the analysis of morphology. 4 patients had bilateral cancer. Therefore, 146 patients with 150 tumors were enrolled into our study. The mean age was 50.2 ± 10.3 years. The mean tumor size was 23.6 ± 11.2 mm. Of all 150 tumors, 117 were infiltrating ductal carcinomas (IDC). Tumors were graded as low in 13 (8.7%), intermediate in 63 (42%), and high in 67 (44.7%), whereas 7 tumors (4.7%) had no an exact grade. 43 tumors (28.7%) were classified as luminal A subtype, 69 tumors (46%) as luminal B subtype, 16 tumors (10.7%) as HER2 subtype, and 22 tumors (14.7%) as TNBC subtype. Ipsilateral axillary lymph node matastasis was confirmed in 48 tumors.

The average score of TC was 55.7% ± 13.5% (TC_1_) measured by the first observer. The second observer obtained an average socre of TC with 57.7% ± 13.0% (TC_2_). A moderate intraclass correlation coefficient (r = 0.826) was found for the measurment of TC by two observers. The average score of TC measured by two observers was 56.7% ± 12.7% (TC_3_). Good agreements were found when TC_1_ and TC_2_ were compared with TC_3_, and the correlation coefficients were 0.957 and 0.954, respectively (Table 3).

**Table 3:** Correlation between two observers and the means-score group of TC.

	*P* value	r value
TC_1_-TC_2_	<0.001*	0.826
TC_1_-TC_3_	<0.001*	0.957
TC_2_-TC_3_	<0.001*	0.954

*P < 0.05.

TC correlated inversely with tumor size (r = −0.224, P < 0.001), ER (r = −0.490, P < 0.001) and PR (r = −0.484, P < 0.001). It also turned to be a positive correlation with Ki67 (r = 0.332, P < 0.001) and histological grade (r = 0.309, P < 0.001), whereas no correlation with paitent age and HER2 (Table 4). In multiple linear regression, the tumor size, ER, PR and Ki67 were independent influential factors of TC.

**Table 4:** Correlation between TC and continuous variables.

	Tumor circularity	
	*P* value	r value
Tumor size	0.006*	−0.224
Patient age	0.835	0.017
ER	<0.001*	−0.490
PR	<0.001*	−0.484
Ki67	<0.001*	−0.332
HER-2	0.228	−0.099
Tumor grade	<0.001*	0.309

*P < 0.05.

**Figure 1:**
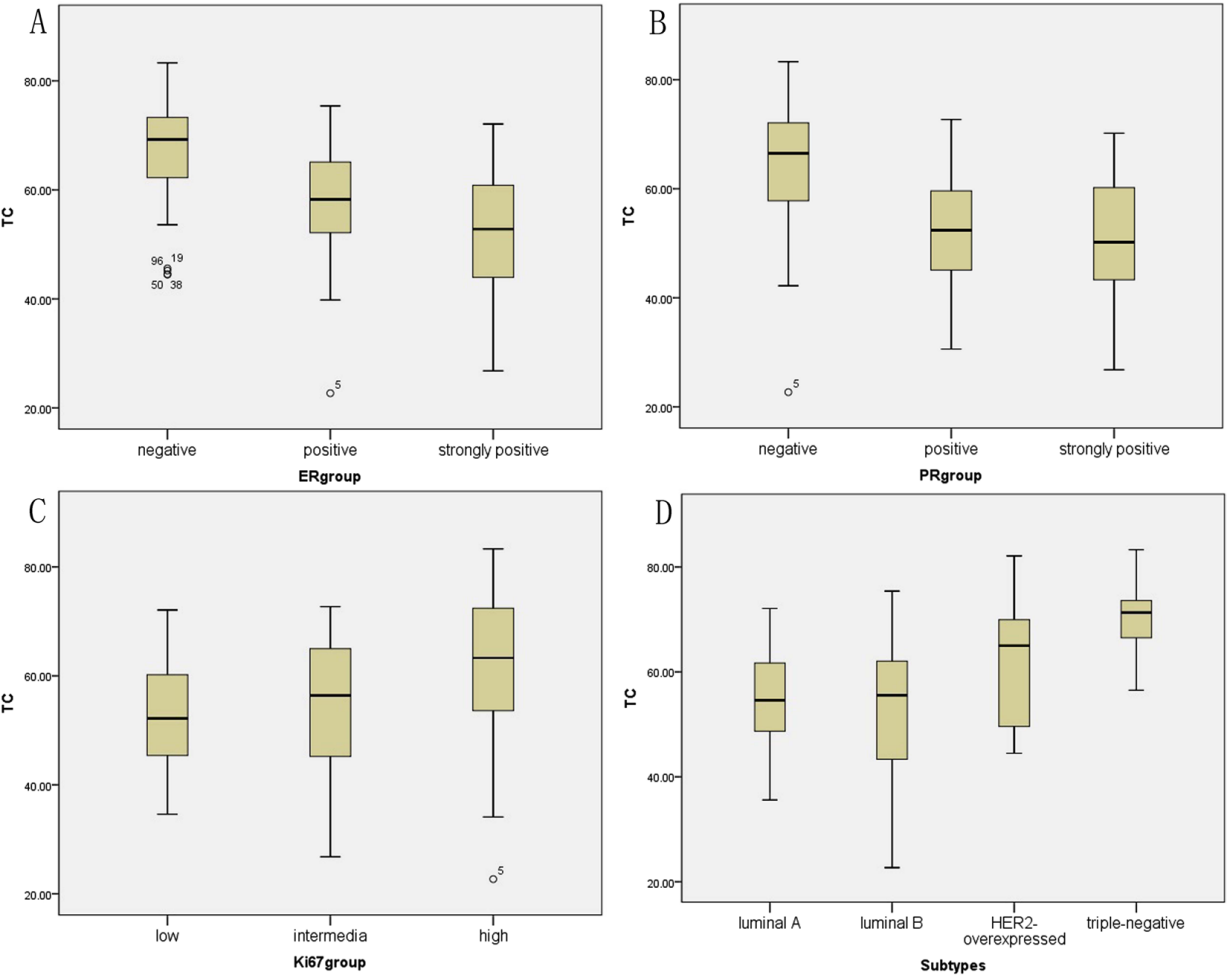
Box plot between TC and subsets in biomarkers and subtypes. (**A**) the negative group showed the highest TC (67%) followed by the positive group (57.1%) and strongly positive group (51.9%). (**B**) the negative group showed the highest TC (64.3%) followed by the positive group (53.1%) and strongly positive group (50.7%) (**C**) the high subset showed the highest TC (62%) followed by the intermediate subset (54.6%) and low subset (52.8%). (**D**) TNBC got the highest TC (70.8%) followed by HER2-overexpressed subtype (61.6%), luminal A (54.7%) and luminal B (52.3%).

TC of all subgroups are listed in Table 5. For ER, the negative group showed the highest TC (67%) followed by the positive group (57.1%) and strongly positive group (51.9%) (Fig. 1A). For PR, the negative group showed the highest TC (64.3%) followed by the positive group (53.1%) and strongly positive group (50.7%) (Fig. 1B). For Ki67, the high subset showed the highest TC (62%) followed by the intermediate subset (54.6%) and low subset (52.8%) (Fig. 1C). For cancer subtype, TNBC got the highest TC (70.8%) followed by HER2-overexpressed subtype (61.6%), luminal A (54.7%) and luminal B (52.3%) (Fig. 1D).

**Table 5:** Correlation between TC and categorical variables.

	N (%)	Tumor circularity	*P* value
		Means, standard deviation	
Histological type			0.997
IDC	117 (78)	56.7 ± 1.2	
Non-IDC	33 (22)	57.0 ± 2.2	
Pathological grade			0.001*
low	13 (8.7)	53.9 ± 3.7	
intermediate	63 (42)	52.8 ± 1.5	
high	67 (44.7)	60.9 ± 1.4	
N/A	7 (4.7)		
axillary lymph node involvement			0.293
positive	48 (32)	58.3 ± 2.0	
negative	102 (68)	55.9 ± 1.2	
Tumor size (mm)			0.012*
<15	25 (16.7)	63.0 ± 2.3	
15~24	64 (42.7)	57.4 ± 1.3	
25~35	43 (28.7)	54.5 ± 2.2	
>35	18 (12)	51.6 ± 3.3	
ER subset			<0.001*
negative	38 (25.3)	67.0 ± 1.7	
positive	28 (18.7)	57.1 ± 2.2	
strongly positive	84 (56)	51.9 ± 1.2	
PR subset			<0.001*
negative	59 (39.3)	64.3 ± 1.5	
positive	41 (27.3)	53.1 ± 1.7	
strongly positive	50 (33.3)	50.7 ± 1.5	
Ki67 subset			0.004*
low	33 (22)	52.8 ± 1.7	
intermediate	66 (44)	54.6 ± 1.5	
high	51 (34)	62.0 ± 1.9	
HER-2 group			0.525
non-expression	57 (38)	58.9 ± 1.8	
low-expression	58 (38.7)	55.2 ± 1.5	
overexpression	35 (23.3)	55.8 ± 2.2	
Molecular subtype			<0.001*
luminal A	43 (28.7)	54.7 ± 1.5	
luminal B	69 (46)	52.3 ± 1.5	
HER2-overexpressed	16 (10.7)	61.6 ± 3.2	
TNBC	22 (14.7)	70.8 ± 1.4	

*P < 0.05.

**Figure 2:**
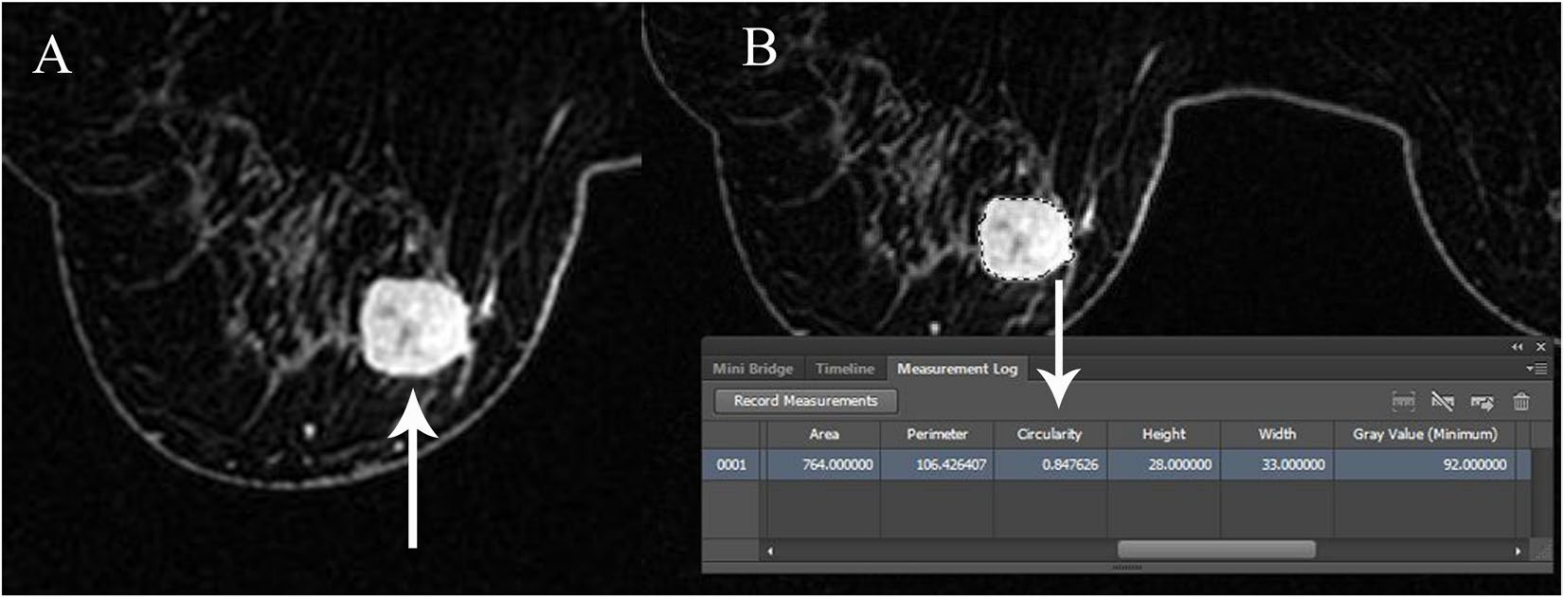
IDC of left breast in a 59-year-old-woman with high grade. IHC stain showed the subtype of TNBC, with the ER score of 0%, PR score of 0%, HER2 negative and Ki67 index of 70%. Axial-T1WI enhanced image showed a 33mm size of round mass with smooth margins (arrow in **A**). TC score is 0.847626 which was calculated automatically by the software Photoshop CS6 (arrow in **B**).

**Figure 3:**
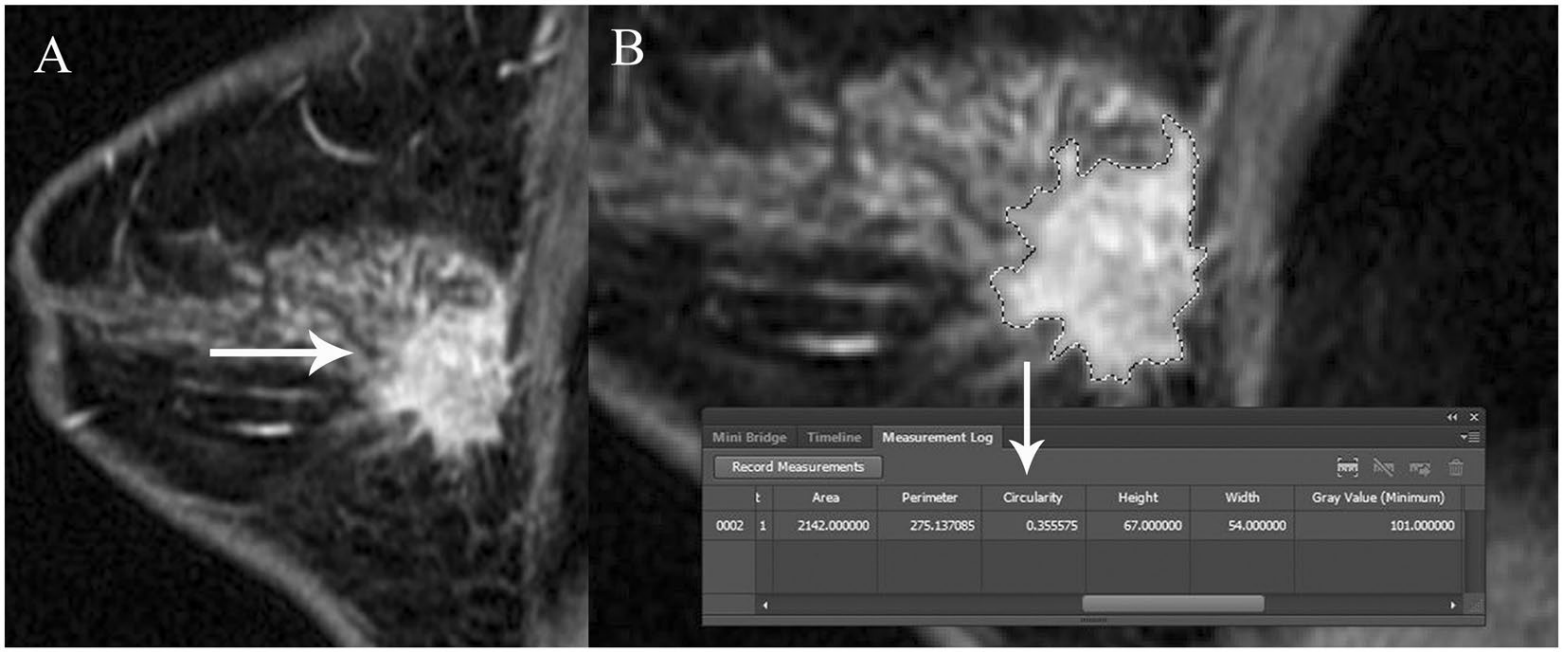
IDC of right breast in a 36-year-old-woman with intermediate grade. IHC stain showed the subtype of luminal B, with the ER score of 90%, PR score of 90%, HER2 score of 1+, and Ki67 index of 80%. Sagittal-T1WI enhanced image showed a 30mm size of irregular mass with spiculated margins (arrow in **A**). The irregular outline of mass can be defined accurately by the image zoom function and then a TC score of 0.355575 was calculated automatically (arrow in **B**).

The between-subgroup analyses of biomarkers and cancer subtypes are listed in Tables 6 and 7, respectively. For ER, significant difference was found in the between-subgroup analysis using one-way ANOVA. For PR, significant difference was found in the between-subgroup analysis except the difference between the positive group and strongly positive group. For Ki67, significant difference was also found in the between-subgroup analysis except the difference between the intermediate group and low group. For cancer subtype, significant difference was found in the between-subgroup analysis except the difference between the luminal A group and luminal B group. The representable cases about TNBC and luminal B subtype have been displayed in Figs 2 and 3, respectively.

**Table 6:** One-way ANOVA analysis with TC and ER, PR, Ki67.

	Tumor circularity	*P* value
	Mean difference, standard error	
ER negative-ER positive	9.8 ± 2.8	0.001*
ER negative-ER strongly positive	14.8 ± 2.2	<0.001*
ER positive-ER strongly positive	5.0 ± 2.4	0.041*
PR negative-PR positive	10.8 ± 2.3	<0.001*
PR negative-PR strongly positive	13.7 ± 2.1	<0.001*
PR positive-PR strongly positive	2.9 ± 2.3	0.217
High Ki67–intermediate Ki67	6.0 ± 2.3	0.009*
High Ki67-low Ki67	8.5 ± 2.7	0.002*
Intermediate Ki67-low Ki67	2.4 ± 2.6	0.354

*P < 0.05.

**Table 7:** One-way ANOVA analysis with TC and subtypes.

	Tumor circularity	*P* value
	Mean difference, standard error	
TNBC-Luminal A	15.8 ± 2.9	<0.001*
TNBC-Luminal B	18.1 ± 2.7	<0.001*
TNBC-HER2-overexpressed	8.7 ± 3.6	0.018*
HER2-overexpressed-Luminal A	7.2 ± 3.2	0.029*
HER2-overexpressed-Luminal B	9.4 ± 3.1	0.003*
Luminal A-Luminal B	2.2 ± 2.2	0.3

*P < 0.05.

## Discussion

The present study indicated that TC correlated inversely with ER and PR. It also turned to be a positive correlation with Ki67 and histological grade. The presence of ER and PR in the cancer cell is important in guiding treatment. Hormone receptor-positive tumors usually have a good prognosis. They are usually less aggressive, lower grade tumors with a lower risk of spreading than hormone receptor–negative ones. Patients with receptor–negative tumors will not be able to respond to hormone therapy, and this can affect their chance of survival^14–16^. Extensive studies had proved that Ki67 was closely relevant to the recurrence and metastasis of breast cancer. The use of Ki-67 as a predictive and prognostic marker in breast cancer has been widely investigated. A breast tumor that scores high for Ki-67 is made of cells that are rapidly dividing and growing. Thus, patients with higher proliferative activity in tumors might require more aggressive therapy and closer clinical monitoring of their disease. Neoadjuvant chemotherapy for breast cancer is considered to be the most practical *in vivo* chemosensitivity test. After neoadjuvant chemotherapy, lower Ki-67 values indicate a better prognosis^17,18^. Our results showed that the higher score of TC, the higher level of Ki67, whereas the lower level of ER and PR. It indicated that tumor morphology was associated with the above three IHC biomarkers of breast cancer. Thus, TC may be a valuable prognostic factor to predict the worse clinical outcomes in patients with breast cancer.

In the present study, TNBC got the highest TC (70.8%) followed by HER2-overexpressed subtype (61.6%), luminal A (54.7%) and luminal B (52.3%). TNBC seems to present as a relatively benign appearance when comparing with other subtypes. This was coincident with the previous researches^19,20^. In our opinion, TC can be used as a quantitive index of MRI morphology to evaluate the subtypes of breast cancer, especially the TNBC.

Some researchers calculated TC by other methods. Bae *et al*.^21^ and Ku *et al*.^22^ obtained tumor roundness by software developed in-house using Microsoft Visual C++. Bae *et al*.^21^ suggest that breast tumors with lower ER expression and higher cellular proliferation or biologically aggressive triple-negative tumors are likely to manifest with relatively benign morphologic features. Ku *et al*.^22^ reported the positive correlation between tumor roundness and tumor-infiltrating lymophocytes. Moon *et al*.^23^ measured the tumor volume and spheroid-ellipsoid discrepancy (CED) by the postoperative specimen. Tumor is nearly a round shape if SED measured closer to zero. The result showed that TNBC got the lowest score of SED. The result of our study are consistent to the above researches. In addition to biomakers, our study also yielded an inverse correlation between tumor size and TC, which was agreeded with the findings of Moon *et al*.^23^. Neverthless, Bae *et al*.^21^ reported none statisitical significance of correlation between tumor size and TC. Thus, further studies about the correlation between tumor size and TC should be warranted.

Base on multiple linear regression, the tumor size, ER, PR and Ki67 were found as independent influential factors of TC. However, pathological subtype was not an independent influential factors of TC. The reason may be the advice of standard grouping had not been unified, even in St.Gallen panel. Clinical validation of Ki67 has proved difficult, while high and low values are reproducible and clinically useful, there appears to be no optimal cut point^12^. It directly due to a elusive subtype of luminal A or luminal B. Using TC to make quantitive analysis with Ki-67 may help to reduce variability because Ki67 displays a continuous distribution^24^.

The association between tumor morphology and IHC or molecular subtypes of breast cancer had been studied using mammography or US in previous researches^25,26^. However, the true size of a cancer is often underestimated on mammography and ultrasound. So the significance of these studies was equivocal. MRI affords the radiologist unique advantages over mammography and ultrasound. The better 3D spatial resolution gives it a better ability to delineate the morphology of a cancer. Furthermore, MRI has a better ability to detect occult, multifocal/multicentric disease and to image both breasts and the chest wall.

The present study had a number of limitations. Firstly, this was a retrospective study with a single-institution database. It may lead to bias and misinterpretation of the results. Secondly, the non-mass lesions were excluded from our study due to the poorly defined boundaries. This also may lead to a bias, although they were only a small proportion of the lesions, especially in TNBC group. Now we are trying to improve the function of software in order to get more accurate identification of tumor outline. If the software can accurately identify the boundaries of non-mass lesions, the limitation will be solved in our future study. Thirdly, the number of patients was relatively small in HER2 and TNBC group due to the low percentage of these two subtypes in breast cancer. The calculation of the sample size to identify a significant effect estimate was absent. Thus, a prospective multiple-institution study with a larger population was needed in the future.

In conclusion, our study suggests that TC can be used as an imaging biomarker to predict the aggressiveness of newly diagnosed breast cancers. TNBC seems to present as an orbicular appearance when comparing with other subtypes.
